# “Reliability of the Norwegian version of the short physical performance battery in older people with and without dementia”

**DOI:** 10.1186/s12877-017-0514-4

**Published:** 2017-06-09

**Authors:** Cecilie Fromholt Olsen, Astrid Bergland

**Affiliations:** Faculty of Health Sciences, Oslo and Akershus University College of Applied Sciences, 0130 Oslo, Norway

**Keywords:** Reliability, SPPB, Physical performance, Dementia, Norwegian

## Abstract

**Background:**

The purpose of the study was to establish the test–retest reliability of the Norwegian version of the Short Physical Performance Battery (SPPB).

**Methods:**

This was a cross- sectional reliability study. A convenience sample of 61 older adults with a mean age of 88.4(8.1) was tested by two different physiotherapists at two time points. The mean time interval between tests was 2.5 days. The Intraclass Correlation Coefficient model 3.1 (ICC, 3.1) with 95% confidence intervals as well as the weighted Kappa (K) were used as measures of relative reliability. The Standard Error of Measurement (SEM) and Minimal Detectable Change (MDC) were used to measure absolute reliability. The results were also analyzed for a subgroup of 24 older people with dementia.

**Results:**

The ICC reflected high relative reliability for the SPPB summary score and the 4 m walk test (4mwt), both for the total sample (ICC = 0.92, and 0.91 respectively)) and for the subgroup with dementia (ICC = 0.84 and 0.90 respectively). Furthermore, weighted Ks for the SPPB subscales were 0.64 for the chair stand, 0.80 for gait and 0.52 for balance for the total sample and almost identical for the subgroup with dementia. MDC-values at the 95% confidence intervals (MDC95) were calculated at 0.8 for the total score of SPPB and 0.39 m/s for the 4mwt in the total sample. For the subgroup with dementia MDC95 was 1.88 for the total score of SPPB and 0.28 m/s for 4mwt.

**Conclusions:**

The SPPB total score and the timed walking test showed overall high relative and absolute reliability for the total sample indicating that the Norwegian version of the SPPB is reliable when used by trained physiotherapists with older people. The reliability of the Norwegian SPPB in older people with dementia seems high, but due to a small sample size this needs further investigation.

## Background

Physical function is a strong biomarker for health in older people [1]. Physical function can be characterized by measures of physical performance, which are objective tests of peoples’ performance of standardized tasks, evaluated according to predetermined criteria that may include counting repetitions or timing the activity. Screening and assessment of physical function among older adults can have several important purposes. It is important that functional decline can be detected early, making it possible to intervene to reverse it or prevent further decline. Furthermore, measurements of physical performance are important outcome measures in studies evaluating the effect of interventions [2, 3].

The short physical performance battery (SPPB) is a commonly used test of physical performance among older populations [2–7]. More accurately, it is a measure of lower-extremity function, consisting of three subtests: standing balance, walking, and rising from a chair. The measure has been shown to predict outcomes such as falls, institutionalization, and death [2, 3, 8, 9]. The validity of the SPPB has been demonstrated in several analyses showing a gradient of risk of admission to a nursing home and mortality along the full range of the scale [3]. Previous research suggest that the SPPB can detect early stages of frailty [10] and that a cut-off score of 9 can discriminate frail from non-frail older adults [10–12]. Frailty is a common and important geriatric syndrome characterized by age-associated declines in physiologic reserve and function across multiorgan systems, leading to increased vulnerability for adverse health outcomes [12]. Furthermore, an SPPB score of ≤10 could be predictive of future decline in mobility [13].

The Norwegian version of the SPPB was translated into Norwegian by Bergh et al. 1 in 2013 [14, 15]. This version has not previously been tested for its reliability. Reliability is the extent to which scores for persons who have not changed are the same for repeated measurements over time. Reliability also indicates the degree of which a test is free of measurement error. Measurement error is the systematic and random error in a patient’s score that cannot be attributed to true changes in the construct to be measured [16]. Test–retest reliability is when the repeated measurements of one person are done by the same rater on two different occasions [16]. It could be argued that the SPPB involves few instructions and therefore translation to a different language is not required. However, this could increase the risk of misinterpretation by the assessor and the person being tested, and subsequently lower the validity and reliability of the tool. There seems to be international consensus that measurement tools should be translated and assessed for non-English speaking populations both for the use in research and in clinical settings [16, 17].

Regarding relative reliability, previous studies have found acceptable to high test–retest reliability for the original English version of the SPPB in U.S populations with ICC-values ranging from 0.81–0.92 [5, 18, 19]. Gomez et al. [20] found an ICC of 0.87 for the total score of the Spanish version of the SPPB used in the Columbian Andes. High relative reliability values have also been found for the SPPB in diverse Brazilian and Canadian populations [4, 21].

Two previous systematic reviews evaluating the psychometric properties of instruments to measure physical performance have concluded that the SPPB is a reliable and valid tool for measuring lower limb strength in the community living elderly [6, 22]. However, due to lack of studies which report the absolute reliability of the SPPB, only the relative reliability has been reviewed. We found only two single studies reporting absolute reliability values of the SPPB; these results were for the original English version and were from a study of older people with mild to moderate loss of function [19, 23]. Perera et al. [23] report a SEM of 1.42 for the SPPB summary score and 0.06 m/s for 4 m walking speed in a mixed older population. Mangione et al. [19] reported a SEM of 1.2 for the SPPB summary score and 0.08 m/s for free gait speed over 2.4 m in older African Americans. Freiberger et al. [6] called for more studies on the absolute reliability of performance based physical function scales for older people, as this property can be an important determinant for use in clinical practice.

Dementia is a general term for a decline in cognitive abilities which interferes with everyday life [24]. The older people get, the higher the prevalence of dementia [24]. Due to their cognitive difficulties, people with dementia may need different approaches when physical performance is being measured, such as for example more time to complete a test and/or a demonstration instead of verbal instructions [25]. Clinical observation of people with dementia often reveals increasing variability of performance with increasing levels of dementia [26]. It is, therefore, particularly interesting to evaluate the reliability of performance measures when used in a population of older people with dementia. Only one previous study has assessed the reliability of the SPPB for older people with dementia [27]. Fox et al. [27] conducted a pilot reliability study of several measurements of physical function including the SPPB with 12 participants with dementia living in aged care facilities. They found acceptable relative reliability, but the absolute reliability was deemed questionable.

To our knowledge, no other study has reported on the reliability of the Norwegian version of the SPPB. The purpose of this study was thus to determine both the relative and the absolute test–retest reliability of the SPPB in Norwegian for a population of frail elderly people. In addition, we performed a separate reliability analysis for a subsample which had been diagnosed with dementia.

## Methods

The participants were tested with the SPPB by the same rater at two different time points. Mean time between test and retest was 2.5 days with a time span of 1–7 days. All tests were conducted between 9 am and 4 pm. The same test room was used for each test and adequate spacing and lighting was assured to ensure optimal test performance. Standardized equipment was used for all the participants. Two experienced physiotherapists who had carefully familiarized themselves with the SPPB test were involved in the study. They used the Norwegian test manual as well as video material from the original test-development as means of preparation. The testers were instructed not to familiarize themselves with the scores on the first test before performing the retest.

A convenience sample of 62 older people were eligible and participated in the study. The participants were recruited from a community center for seniors in Oslo, Norway. Among the participants, 39 were inpatients/living in a nursing home adjacent to the senior center, and 22 participants lived at home and attended the senior center on a weekly basis. Twenty-four of the nursing- home residents had been diagnosed with dementia, based on a comprehensive geriatric assessment as confirmed by the nursing home’s physician. The recruitment was a targeted recruitment at the senior center in the form of a short talk on the study aims. The inclusion criteria were: being aged 67 years or older and being able to stand up alone or with the help of one person and being able to walk six meters with or without a walking aid. The exclusion criteria were: patients who were medically unstable or had severe communication problems. Further details about the participants can be found in Table 1 **.**

**Table 1:** Baseline characteristics and SPPB summary score at test 1 and 2 for all participants and for group comparison dementia/no dementia

Variable^a^	All participants *N* = 61	Participants with dementia *n* = 24	Participants without dementia *n* = 37	*P*-value
Age	88.4 (8.1), (67–102)	88.3 (6.2), (69–97)	88.4 (9.2) (67–102)	.958^e^
Sex				
Women	50 (82)	21 (87.5)	29 (78.4)	.572^f^
Men	11 (18)	3 (12 .5)	8 (21.6)	
Use of walking aids				
*Frame/rollator*	36 (59.0)	13 (54.2)	23(62.2)	.307^f^
*Cane*	3 (4.9)	0	3 (8.1)	
*Other*	6 (9.9)	3 (12.5)	3 (8.1)	
*None*	16 (26.2)	8 (33.3)	8 (21.6)	
Type of dwelling				
*Nursing Home*	39 (63.9)	24 (100)	15 (40.5)	.001^f^
*Home*	22 (36.1)	0	22 (59.5)	
Number of days between tests	2.5(1.5), (1–7)	2.5 (1.3), (1–6)	2.5 (1.6), (1–7)	.972^f^
SPPB^b^ summary score test 1	3.7 (2.4), (0–10)	2.2 (1.4), (0–4)	4.7 (2.4), (1–10)	.001^e^
SPPB^b^ summary score test 2	4.1 (2.5), (0–9)	2.4 (2.0), (0–7)	5.2 (2.1), (1–9)	.001^e^
Gait speed m/s	0.47 (0.17) (0.13–0.97)	0.40 (0.16) (0.13–0.79)	0.51 (0.17) (0.22–0.97)	.02^e^

^a^Continuous variables are expressed in mean (SD), (min-max), categorical variables are expressed in number (%).

^b^Short Physical Performance Battery (SPPB), min-max = 0–12, higher score indicates better function

^e^Independent sample t-test ^f^Chi-Square test

Participants were asked between tests if they had experienced illness or other events that could affect the results on the second test. None of the participants reported such an event.

The Norwegian version of SPPB which was translated into Norwegian by Bergh et al. [14] was used in the study. This test consists of two scoring sheets in which the first sheet is used for absolute values measured in seconds and the other sheet is used for comments and scoring according to the test’s 0–4-point scale.

Five performance scores (from 0 to 4) were given for each test, with a score of 0 representing inability to complete the test and 4 the highest level of performance. For tests of standing balance, the subjects were asked to attempt to maintain their feet in the side-by-side, semi-tandem (heel of one foot beside the big toe of the other foot), and tandem (heel of one foot directly in front of the other foot) positions for 10 s each. The subjects were given a score of 1 if they could hold a side-by-side standing position for 10 s but were unable to hold a semi-tandem position for 10 s, a score of 2 if they could hold a semi-tandem position for 10 s but were unable to hold a full tandem position for more than 2 s, a score of 3 if they could stand in the full tandem position for 3 to 9 s, and a score of 4 if they could stand in the full tandem position for 10 s.

A 4 m (13 ft) walk at the subjects’ habitual pace was timed, and the participants were scored according to quartiles for the length of time required. The time of the faster of two walks was used for scoring.

Subjects were asked to fold their arms across their chests and to stand up from a sitting position once; if they successfully rose from the chair, they were asked to stand up and sit down five times as quickly as possible. Quartiles for the length of time required for this measure were used for scoring. The summary performance score was created by adding the scores for the tests of standing balance, walking, and repeatedly rising from a chair giving a maximum score of 12.

The scoring protocol for the SPPB includes comments regarding performance and the reasons for not completing an item. In the Norwegian version, a meters/s calculation for walking, as well as an alternative test for sit-to- stand (STS) where the person is allowed to rise and sit with the use of chair handles, has been added as an appendix. This is not a modification of the SPPB as such, since none of these additions are scored on a scale of 0–4 or added to the summary score of the SPPB [14].

### Statistical Analysis

Data was analyzed using the SPPS 20.0 for Windows (IBM Corporation, Armonk, NY, USA). Sample characteristics are presented in means and standard deviations (SD) for continuous variables and numbers and percentages for categorical variables. There were missing data for the retest walk test for one of the participants and this score was left missing in the analysis.

The Cronbach Alpha was used to assess the internal consistency of the test. Cronbach Alpha values are considered excellent if higher than 0.9, moderate at 0.8 and 0.7 and low if less than 0.7 [28]. Internal consistency was also tested with the Inter-Items Correlation since Cronbach Alpha is sensitive to the number of items in a scale [29]. An optimal range for the inter-item correlation is 0.2 to 0.4 [29].

**Relative** reliability was assessed using the intraclass correlation coefficient (ICC) 3.1 (2-way mixed-model single measure) with 95% Confidence Intervals (95% CIs). ICC values range from 0 to 1 where 1 corresponds to perfect agreement. An ICC of 0.80 or higher was considered high, 0.60–0.79 moderate and less than 0.60 was considered to be poor relative reliability [28, 30, 31].

The SPPB produces both categorical and continuous data. Test–retest agreement on individual items of the SPPB was analyzed with linear weighted κ analyses. The weighted κ score measures the agreement of test–retest, adjusted for the amount of agreement expected by chance and the magnitude of disagreement [32]. A K value of 0.75 or higher indicates excellent agreement, between 0.74 and 0.4 indicates fair to low agreement and less than 0.4 indicates poor agreement [33]. Weighted K was calculated in Excel for Windows 8 with the Real Statistics Resource Pack. Bland-Altman plots were plotted to demonstrate the 95% limits of agreement.

**Absolute** reliability was assessed by the standard error of measurement (SEM) and minimal detectable change (MDC). The SEM and MDC are presented in the unit of the test score making it easier to interpret and use the results in the clinic [16]. MDC is calculated from the SEM and represents the smallest change in a score that, with *P* < 0.05, can be interpreted as real change and not measurement error [16]. SEM was calculated by the following formula: SEM = SD √(1–ICC) [30]. MDC was calculated as SEM × 1.96 × √2 [16].

Since the SPPB Norwegian version includes calculations of gait-speed in meters/s, [14] and since all participants were able to perform this test, we chose to conduct a separate reliability test of the 4-m walk test (4mwt) measured in m/s.

The floor and ceiling effects were calculated as the percentage of the sample scoring the minimum of the maximum of the possible summary score. Floor and ceiling effects of more than 15% is considered significant [16, 34]. The magnitude of the floor and ceiling effects can be used to indicate the sum score’s ability to discriminate between subjects [16].

Regarding power, current literature on reliability recommends a minimum of 55 participants for reliability studies [16, 35]. We also conducted a power analysis based on a desired reliability coefficient of 0.90 as demonstrated in previous research and a minimum coefficient of 0.80 [6, 18]. With a one-sided 95% CI and 2 testing sessions and with an alpha level of .05, a minimum sample size of 46 was required [36, 37].

### Ethical considerations

The study was approved by the Regional Committee for Medical Ethics in south-east Norway. The principal caregiver gave written and verbal information about the study to the patients and their relatives. All the participants gave their own written consent (or a relative consented on their behalf) to participation in the study, and they were informed that they could refuse or withdraw participation at any stage in the study.

## Results

The study had only one drop out, due to sudden death**.** Sample characteristics for the total sample (*N* = 61) as well as divided by group, dementia (*n* = 24) /not dementia (*n* = 37), are presented in Table 1 **.** Participants were predominantly female (*n* = 50, 82%), the mean age of the participants was 88.4 (range 67–102 years). Furthermore, regarding age, 45.9% (*n* = 28) of the sample were 90 years of age or older, and 64% lived in a nursing home. Regarding walking aids, 16 did not use any walking aids, 36 used a rollator and 3 walked with a cane.

Twenty-four participants had a diagnosis of dementia, and all 24 of them lived in a nursing home. Among the participants without dementia, 22 lived in their own home and 15 in a nursing home. There was no significant difference between the two groups regarding the sample characteristics: sex, age, use of walking aids, and number of days between tests. There was, however, a statistically significant difference between the two groups with regards to type of dwelling, SPPB summary score, and mean walking speed (see Table 1). The group with dementia had a statistically significant lower summary score on the SPPB with a maximum score of 4 on the first test and 7 on retest. Mean walking speed was 0.40 m/s for the group with dementia and 0.51 m/s for the group without dementia (*n* = 37).

Regarding the SPPB summary score, average score on Test 1 was 3.7 (2.4) and on Test 2 4.1 (2.5). Score range on Test 1 was 0–10, 6.6% of the sample scored the lowest score 0 and 1.6% scored 10. Score range on Test 2 was 0–9, 8.2% scored 0 and 3.3% scored 9. This means that there was no floor and ceiling effect for the SPPB summary score either on test or retest. On Test 1 85.2% scored 6 or less, on Test 2 80.3% scored 6 or less.

Table 2 presents the distribution of scores on the test and retest for the individual subscales of the SPPB. The table shows the number of participants with a score of zero, one, two, three and four on each item. On the balance subscale 27.9% of the sample scored 0 points on both test and retest, on the STS item 59% scored 0 points on Test 1 and 49% scored 0 points on Test 2. Hence, there was a floor effect on both these items.

**Table 2:** Distribution of participants’ scores (0–4) on test (T1) and retest (T2) of the individual subscales of the SPPB

SPPB Item	0 Points		1 Point		2 Points		3 Points		4 Points	
	T1	T2	T1	T2	T1	T2	T1	T2	T1	T2
Balance subscale	17	17	18	11	14	19	8	11	4	3
Walking subscale	6	5	25	23	13	15	16	14	1	4
STS^a^ subscale	36	30	17	21	2	7	5	3	1	0

^a^Sit to stand (STS) subscale of the Short Physical Performance Battery (SPPB)

Regarding internal consistency, the Cronbach Alpha coefficient of the SPPB was 0.63 for test 1 and 0.66 for Test 2. The inter-item correlation mean was 0.36 (0.27–0.41) and 0.41 (0.39–0.42) for Tests 1 and 2 respectively.

Table 3 presents the results from the relative and absolute reliability analysis. The ICC reflects high reliability for the summary score of the SPPB (ICC = 0.92), indicating that there was no systematic error in the measurements. The ICCs for the SPPB subtests and the 4mwt measured in m/s were also high (ICCs ranging from 0.82 to 0.95). However, as presented in Table 4 **,** the Weighted Kappa score for the three subtests was 0.52, 0.80, and 0.64 respectively, suggesting fair to low agreement for balance and STS and excellent agreement for the walking subscale.

**Table 3:** showing the mean, min-max scores, ICC, SEM and MDCs for SPPB subscales and summary score and 4mw for the total sample (*N* = 61)

Test item	Test 1 mean (SD) min-max	Test 2 Mean (SD) min-max	ICC	95% CI	SEM	MDC95	MDC90
Balance subscore	1.4 (1.2)	1.5 (1.2)	0.82	0.70–0.89	0.51	1.4	1.2
	0–4	0–4					
Walking subscore	1.7 (1.0)	1.8 (1.1)	0.95	0.91–0.97	0.23	0.6	0.5
	0–4	0–4					
STS subscore	0.7 (1.0)	0.7 (0.9)	0.83	0.72–0.90	0.39	1.1	0.9
SPPB summary score	3.7 (2.4)	4.1 (2.5)	0.92	0.88–0.95	0.28	0.8	0.7
	0–10	0–9					
4mwt1 m/s	0.47 (0.17)	0.48 (0.17)	0.91	0.85–0.95	0.14	0.39	0.33
	0.13–0.97	0.14–0.81

**Table 4:** Test-retest Weighted Kappa and 95% CI for total sample and divided by diagnosis

Item	Total sample (*N* = 61)	95% CI	Dementia (*n* = 24)	95% CI	Not dementia (*n* = 37)	95% CI
Balance subscore	0.52(0.07)	0.37–0.66	0.40(0.10)	0.20–0.60	0.46(0.10)	0.26–0.66
Walking subscore	0.80(0.05)	0.71–0.89	0.88(0.07)	0.74–1.0	0.73(0.07)	0.59–0.86
Sit to stand subscore	0.64(0.07)	0.50–0.78	0.60(0.17)	0.26–0.94	0.59(0.09)	0.41–0.77

**Fig. 1:**
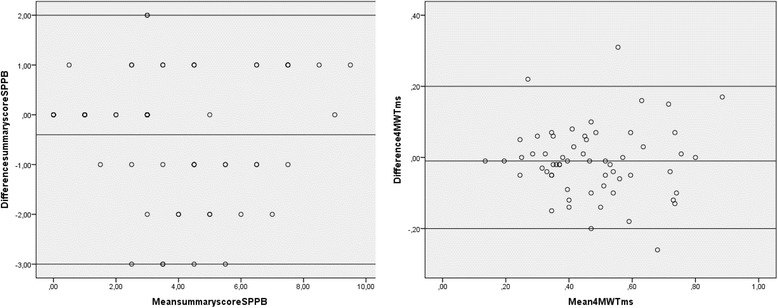
Bland-Altman plots for the Short Physical Performance Battery summary score and 4 m walk test (*N* = 61). (Short Physical Performance battery sum score: min-max 0–12, 4 m walk test measured in meters/s)

As shown in Fig. 1, no systematic variability was demonstrated in the Bland-Altman plot with 95% limits of agreement between tests being −3 to 2 points for the SPPB summary score and −0.2 to 0.2 m/s for the 4mwt. Mean difference between tests was −0.4 points for the SPPB summary score and −0.01 m/s for the 4MWT.

The SEM shows the test–retest differences in absolute values, using the same unit as the measurement of interest. The MDC values show the limits of change a participant has to achieve before we can say that the change is a clinical change beyond measurement error. In Table 3, both the MDC95 and the MDC90 reflecting a 95% and 90% certainty, are presented.

For the SPPB summary score SEM was 0.28, MDC95 was 0.8 and MDC90 was 0.7. For 4mwt in m/s SEM was 0.14, MDC95 0.39; and MDC90 0.33.

Regarding the subgroup analyses of dementia/no dementia, presented in Table 5 **,** there was a slight difference in SPPB summary score ICCs with 0.84 for dementia and 0.91 for no dementia. For the other scores, ICCs and weighted Ks were almost identical in the two groups. In other words, relative reliability was only slightly lower for the group with dementia. Likewise, absolute reliability values of SEM and MDCs did not differ between groups for the total score of SPPB (SEM = 0.68, MDC95 = 1.88, MDC90 = 1.59). The greatest difference in absolute reliability was found for the STS subscale and for the 4mwt in favor of the group with dementia. For example, for the 4mwt the estimated SEMs for the group without dementia were almost double the value of the group with dementia (SEM = 0.18 and 0.10 respectively and MDC95 = 0.50 and 0.28 respectively).

**Table 5:** showing the mean scores, ICC, SEM and MDCs for SPPB subtests, SPPB summary score and 4mwt for the sample split by dementia/no dementia diagnosis

Group	Dementia diagnosis *n* = 24							No dementia diagnosis *n* = 37						
Test item	Test 1^a^	Test 2^a^	ICC	95% CI	SEM	MDC95	MDC90	Test 1^a^	Test 2^a^	ICC	95% CI	SEM	MDC95	MDC 90
Balance subtest	0.8(0.9)	0.8(1.0)	0.74	0.38–0.89	0.48	1.33	1.12	1.8(1.2)	2(1.1)	0.79	0.59–0.89	0.53	1.47	1.24
	0–3	0–3						0–4	0–4					
Walking subtest	1.2(1.0)	1.3(0.9)	0.96	0.92–0.98	0.19	0.53	0.44	2(0.9)	2.2(1.0)	0.92	0.84–0.96	0.27	0.75	0.63
	0–3	0–3						1–4	1–4					
STS^b^ subtest	0.2(0.4)	0.3(0.6)	0.83	0.72–0.90	0.21	0.58	0.49	1(1.0)	1(0.9)	0.82	0.64–0.91	0.40	1.11	0.93
	0–1	0–2						0–4	0–3					
SPPB sum score	2.2(1.4)	2.4(2.0)	0.84	0.64–0.93	0.68	1.88	1.59	4.7(2.4)	5.2(2.1)	0.91	0.81–0.95	0.68	1.88	1.59
	0–4	0–7						1–10	1–9					
4 mwt^c^ m/s	0.40 (0.16)	0.40 (0.17)	0.94	0.85–0.97	0.10	0.28	0.23	0.51 (0.17)	0.53 (0.16)	0.88	0.77–0.94	0.18	0.50	0.42
	0.13–0.79	0.14–0.75						0.22–0.97	0.25–0.81					

^a^values are presented as mean (SD), minimum-maximum

^b^Sit to Stand subscale.

^c^4-meter walking test measured in meters/s.

## Discussion

The findings of the current study showed a substantial agreement and overall a very good relative reliability for the use of the Norwegian version of the SPPB in a population of older people. Our results regarding absolute reliability were somewhat different to previous research both for the total sample and for the subsample with dementia in that, overall, we found lower SEM and MDC values.

It must be considered that we had reached a sample of older people with a high mean age (88.4 years) and a low physical function as reflected in the very low mean SPPB total score (4 points). The baseline summary SPPB score both for those with and without dementia were 2.2 and 4.7 respectively, indicating severely limited function [3, 5]. The scores are well below the 9 and 10 that have been proposed as cut-off scores indicating frailty and mobility restrictions respectively [10–13]. The mean gait speed, which was 0.47 m/s for the total sample, provides further proof of the limited level of function. A gait speed of <0.6 m/s in the 4mwt is considered a cut-off for identifying persons with deteriorating health and physical function [38]. Furthermore, the lower mean walking speed in the dementia subsample (0.40 m/s) is consistent with previous studies that have shown negative associations between walking speed and cognitive function [39–41].

The majority of the other studies to which we have compared our findings have included older people with better physical function [4, 6, 13, 19, 20, 22, 23, 27, 42–45]. For example, Fox et al. [27] found a mean SPPB summary score of 4.5 in a group of nursing-home residents with dementia, which was twice as high as for the subsample with dementia in the current study sample.

Previous studies have indicated that there might be ceiling effects of the SPPB in samples of community living elderly [45, 46], and floor effects in the elderly with very low levels of function. However, despite the very low function of our sample, we did not find a floor effect in the summary score of the SPPB. There were, however, obvious floor effects in the subscales relating to balance and sit-to-stand.

### Relative reliability

The ICCs in the current study were overall high and ranging from 0.82 to 0.95 for the total sample and 0.74 to 0.96 for the subgroup with dementia. In comparing the two subgroups with and without dementia, we found there were only slight differences in ICCs and that the relative reliability was overall high for both groups.

Our findings for the total sample comply with other reliability studies of the SPPB [4, 6, 18, 20–22, 43]. Freire et al. [4] found high ICCs both for the population in Quebec (ICC = 0.89) and Brazil (ICC = 0.83). This was for the SPPB total score. The systematic review by Mijnarends et al. [22] reported ICCs of 0.88–0.92. and Kappa values of 0.38–0.95. These reported Kappa values, however, were not weighted, and thus do not take into account the amount of agreement expected by chance and the magnitude of the disagreement [32]. The weighted K results of this current study, we would argue, are therefore more accurate. It should be considered, that somewhat different statistical analyses have been used in the various studies to which we have compared our findings [2–4, 6, 12, 13, 22, 23, 27, 43–45].

We only found one previous study with which we could compare the reliability results for the subsample with dementia. Fox et al. [27] found similar relative reliability results to ours in a very similar sample of older people with dementia living in a nursing home.

Regarding the three subscales of the SPPB, the balance item received the lowest ICC and weighted Kappa and the gait speed subscale received the highest. This concurs with previous research [4, 20, 43]. Measurement of habitual gait speed is widely used and studied in geriatric literature, where it has proven reliable and can be used to predict several adverse health outcomes [47]. Regarding walking distance, original studies on the SPPB [2, 3, 6, 22] were based on the 8 ft. (2.4 m) walk which was later changed to a 4-m walk. In the current study, we tested the reliability of the 4-m walk test. The systematic review by Freiberger et al. [6] criticizes the creation and use of modified versions of the SPPB and we have, therefore, been careful to use the official Norwegian translated version which was translated using the recommended method [14].

The high reliability of the gait speed subscale has led to a discussion about the value of the total SPPB versus gait speed test alone. However, previous research suggests that SPPB might be more sensitive to functional decline than gait speed [5, 10, 12]. For example, a study by Verghese and Xue [10] showed that the SPPB was able to detect early stages of frailty, even among older adults with normal walking speed, indicating that slowing of gait may occur later in the process towards frailty [10]. Furthermore, a multi-dimensional measurement, such as the SPPB, is generally more robust than single item measures; it provides a broader level of assessment and can be used to establish interventions from different functional domains [6]. Cesari et al. [48] point out that either one of the three SPPB subscales may be used separately and still give a good prediction of adverse health events. However, the predictive value seems to increase with a greater number of tests. The clinician must, therefore, measure the value of this increased predictive ability against the greater complexity of administering all three tests in a clinical setting [48].

### Absolute reliability

Because absolute reliability is expressed in the same units as the measurement of interest, the values are easy to interpret in clinical practice. The values obtained in our study can, for example, be used to assess whether changes in lower extremity function after an exercise regimen are due to a real change and not due only to measurement error. For example, we found an MDC of 0.8 for the SPPB summary score, which in practice means that a difference of 1 point on the SPPB would be sufficient to know that measurement error has been exceeded.

There is a general lack of information regarding absolute reliability for the SPPB in earlier publications, and we have few studies with which we can compare our findings [6, 22, 27]. The estimates of absolute reliability for the total sample in this current study are considerably different to those of Perera et al. [23]. They found a SEM of 1.42 for the SPPB and 0.06 m/s for the 4mwt. Using the standard calculation of MDC [16], this would give an MDC95 of 3.9 points for summary SPPB and of 0.17 m/s for 4mwt. We found a much smaller SEM and MDC95 for the summary SPPB (0.28 and 0.8 respectively), but considerably higher values for the 4mwt. It must be noted that Perera et al. [23] also presented a small detectable change of 0.5 points and a so-called “substantial change” of 1 point for the SPPB summary score using anchor-based methods. Even though the latter estimates are based on different statistical analyses, they are closer to our results. Similarly, the results of Mangione et al. [19] show a higher SEM of 1.2 points and an MDC90 of 2.9 points for SPPB summary score and a considerably lower SEM and MDC90 for gait speed (0.08 m/s and 0.19 m/s respectively) compared to the current study. The most plausible explanation for these discrepancies is the difference in sample characteristics and baseline variability. Both these articles present analyses from samples of higher functioning older adults. Perera et al. [23] point out that their results are most relevant in older people with mild to moderate mobility difficulties. The sample in Mangione et al. [19] had a mean baseline score of 8.3 on the SPPB, which is twice the size of that of this current study. Judging by the baseline data presented in these articles, there was also a larger baseline variability in both these studies’ samples compared to ours. This would produce higher SEMs [49].

Regarding dementia, however, using different statistical analysis, Fox et al. [27] found only somewhat higher SEM and MDC values compared to us. They concluded that the absolute reliability values for the SPPB in elderly people with dementia is questionable. In our study, however, we found overall small differences in the SEM and MDCs for the subscales in the group with dementia compared to those without dementia. The SEM and MDCs for the SPPB summary score was identical to the no-dementia subsample (SEM = 0.68 and MDC95 = 1.88). For the 4mwt the absolute reliability came out better than for the no-dementia group and the total sample. It should be remarked, that the study by Fox et al. [27] differs in some respects to ours. Their study had a sample of 12 participants whereas the sample with dementia in our study was twice that size (*n* = 24). The number of days between test and retest was higher in the Fox et al. [27] study (7 days) whereas we had a mean of 2.5 days (maximum 6 days) between tests. Fox et al. [27] also used the original 2.4 m walk instead of the 4 m walk test, which is currently the SPPB standard.

Regarding absolute reliability for walking speed in the group with dementia, Ries et al. [26] found a SEM of 0.06 m/s for 4.5-m gait speed (measured with GAITRite Mat), that is a somewhat lower, but comparable, SEM to that which we found. It is worth noting that Ries et al. [26] also stratified their sample by dementia severity level and found no difference in reliability of gait speed between those with mild to moderate dementia and those with severe dementia. Likewise, in our sample, having a dementia diagnosis does not seem to have a negative effect on the reliability of the gait speed measurements.

### Internal consistency

Regarding internal consistency, other studies have found Cronbach Alpha (CA) values above the ones found in this study [3, 6]. The issue of CA sensitivity to item numbers has not been highlighted in other studies. We found relatively low CAs, but we also chose to calculate the inter-item correlation which indicated good internal consistency for both test and retest in this study [29]. Assessing the internal consistency of the SPPB can be challenging. It is a multi-dimensional test comprised of three different components of physical function: balance, walking, and strength. These three components might well represent three different constructs within the one broader umbrella-construct of *physical performance* or *lower-extremity physical function*.

### Limitations

The study has some limitations on its generalizability. First, it was performed by two different physiotherapists in a single urban setting. Second, most of the participants were women. The sample had a high mean age and did not score the full range of scores on the SPPB. This also has some statistical consequences in that the SEM is affected by the underlying variability of the scorings [49] . The variability in the current sample was small which could also have resulted in the smaller SEM and MDC values compared to the limited previous research on these estimates [23, 27]. Furthermore, it is unfortunate that we did not collect information regarding the dementia severity level among the participants who had a diagnosis of dementia.

Regarding sample size, the total sample size may seem small, but was well above our statistical sample size estimation. Also, 50–99 is considered a good sample size according to the Cosmin checklist [34]. The sample size for the dementia subgroup, however, is a little below the ideal. Results from a lower sample size are valid, but more uncertain and with an increased risk of type II error [29]. With a lower statistical power, one might find results that are clinically relevant, but are not statistically significant. Results from the dementia subsample analysis should be interpreted with this in mind.

### Implications for practice

#### No other studies have assessed the reliability of the Norwegian version of the SPPB.

The SPPB is frequently used in geriatric settings in Norway [14, 15] . Our study shows that the test–retest reliability of this scale is high in a sample of older people with low physical function in an urban setting. The Norwegian version of the SPPB also appears to be reliable for use with older people with dementia, but this requires further research. More research is also needed regarding other quality criteria of the Norwegian version of the SPPB. For example, a summary score MDC of 0.8 which in practice means that a change of 1 point in the SPPB would be sufficient to know that measurement error has been exceeded, does not necessarily mean that a change of 1 point is also meaningful to patients in a clinical setting. This is rather an issue of the interpretability of the test, a property not explored in this study [16]. However, this study shows promising results regarding the use of the Norwegian version of the SPPB in clinical practice and research relating to older people.

## Conclusions

The Norwegian version of the SPPB appears to have high relative and acceptable absolute reliability as well as good internal consistency when used by trained physiotherapists in a population of older people with and without dementia.

## Abbreviations

4mwt: 4-m walking test
CA: Chronbach alpha
CI: Confidence interval
ICC: Intraclass correlation coefficient
K: Kappa
m/s: Meters per second
MDC: Minimal detectable change
MDC90: Minimal detectable change at the 90% confidence interval
MDC95: minimal detectable change at the 95% confidence interval
SD: Standard deviation
SEM: Standard error of measurement
SPPB: Short physical performance battery
STS: Sit to stand

## Footnotes

1 The Short Physical Performance Battery was translated into Norwegian by Bergh S, (Research Centre for Old Age Psychiatry, Innlandet Hospital trust), Lyshol H (Norwegian Institute of Public Health), Selbæk G (Research Centre for Old Age Psychiatry, Innlandet Hospital trust), Strand BH (Norwegian Institute of Public Health), Taraldsen K and Thingstad P (Research group of geriatrics, St. Olav’s Hospital/Trondheim University Hospital and the Norwegian University of Science and technology). It is available online at: http://legeforeningen.no/Fagmed/Norsk-geriatrisk-forening/Nyheter/2013/SPPB-pa-norsk/
